# Identification of regenerating island-derived protein 3E in dogs

**DOI:** 10.3389/fvets.2022.1010809

**Published:** 2022-10-28

**Authors:** Laureen M. Peters, Judith Howard, Tosso Leeb, Meike Mevissen, Rolf Graf, Theresia Reding Graf

**Affiliations:** ^1^Department of Clinical Veterinary Medicine, Clinical Diagnostic Laboratory, Vetsuisse Faculty, University of Bern, Bern, Switzerland; ^2^Department of Clinical Research and Veterinary Public Health, Institute of Genetics, Vetsuisse Faculty, University of Bern, Bern, Switzerland; ^3^Division of Veterinary Pharmacology and Toxicology, Department of Clinical Research and Veterinary Public Health, Vetsuisse Faculty, University of Bern, Bern, Switzerland; ^4^Department of Surgery and Transplantation, Pancreas Research Laboratory, University Hospital Zürich, University of Zürich, Zürich, Switzerland

**Keywords:** biomarker, canine, *Canis lupus familiaris*, gastrointestinal, pancreas, pancreatitis, sepsis

## Abstract

Regenerating islet-derived protein (REG) 1A (aka pancreatic stone protein) and REG3A (aka pancreatitis-associated protein) are upregulated in humans with sepsis, pancreatitis, and gastrointestinal diseases, but little is known about this protein family in dogs. Our aim was to identify REG1 and REG3 family members in dogs. REG-family genes were computationally annotated in the canine genome and proteome, with verification of gene expression using publicly available RNA-seq data. The presence of the protein in canine pancreatic tissue and plasma was investigated with Western blot and immunohistochemistry, using anti-human REG1A and REG3A antibodies. Protein identity was confirmed with mass spectrometry. Two members of the *REG3* subfamily were found in the canine genome, *REG3E1* and *REG3E2*, both encoding for the same 176 AA protein, subsequently named REG3E. Anti-human REG3A antibodies demonstrated cross-reactivity with the canine REG3E protein in pancreas homogenates. In canine plasma, a protein band of approximately 17 kDa was apparent. Mass spectrometry confirmed this protein to be the product of the two annotated *REG3E* genes. Strong immunoreactivity to anti-human REG3A antibodies was found in sections of canine pancreas affected with acute pancreatitis, but it was weak in healthy pancreatic tissue. Recombinant canine REG3E protein underwent a selective trypsin digestion as described in other species. No evidence for the presence of a homolog of REG1A in dogs was found in any of the investigations. In conclusion, dogs express REG3E in the pancreas, whose role as biomarker merits further investigations. Homologs to human REG1A are not likely to exist in dogs.

## Introduction

Acute pancreatitis and sepsis are two relatively common diseases in dogs, which are potentially fatal if diagnosis and treatment are delayed (1, 2). Laboratory testing is crucial in the diagnosis of both disorders, but a gold standard assay for a rapid and reliable diagnosis is lacking (3–5).

In people, pancreatic stone protein (PSP) has gained attention in the last decade as an early and reliable marker for sepsis, outperforming established inflammatory markers (6–8). This protein was first discovered in pancreatic stones of people with chronic pancreatitis (9). Later, a gene homologous to PSP was discovered in regenerating islets of rats following incomplete pancreatectomy (10). A similar protein was independently discovered in the pancreas of rats with induced acute pancreatitis and named pancreatitis-associated protein (PAP) (11), as well as in tissue of human hepatocellular carcinomas, intestine, and pancreas, thus also termed hepatocarcinoma-intestine-pancreatic protein (12). Later, both PSP and PAP were attributed to the regenerating islet-derived protein (REG) family as REG1A and REG3A, respectively (13).

The REG protein family is a group of C-type lectins, which are approximately 16 kDa in size, containing an N-terminal trypsin cleavage site. Upon digestion with trypsin, the remaining fragments form insoluble fibrils (14). In humans, five members of the REG family, notably REG1A (PSP), REG1B, REG3A (PAP), REG3G, and REG4 exist, whereas mice also have a Reg2 subfamily and two additional members of the Reg3 subfamily (15).

Despite extensive research in the last four decades, the main function of regenerating proteins remains unknown. Besides sepsis, serum levels of REG1A and REG3A are increased in people with pancreatitis (16–18), as well as gastrointestinal pathologies, such as inflammatory bowel disease (19) and colorectal carcinoma (20). Furthermore, a bactericidal effect has been attributed to REG3A and REG3G in the intestinal lumen (21, 22). In the nervous system, threads of REG1A and REG3A were found within neurofibrillary tangles and senile plaques of people with Alzheimer's disease (23), and more recently, REG3A has gained significance as an early marker for cystic fibrosis in neonates (24).

To date, little is known about REG proteins in domestic animals. Pancreatic thread protein, later attributed to the REG protein family, was purified from the pancreas of cows in the eighties (25, 26). In 1991, Bernard et al. demonstrated the presence of a protein of similar size to the human REG-proteins, cross-reacting with human anti-REG1A antibodies in pancreatic juice or tissue from a dog, a pig, a rat, a cow, and a baboon (27). A homolog of REG3A was found in the intestinal mucosa of piglets (28, 29) as well as in Peyer's plaques of the ileum and jejunum of lambs (30). Recently, REG3A was found to be upregulated in fecal samples of dogs with chronic enteropathies compared to healthy control dogs (31). To our knowledge, no further investigations into the REG family have been pursued in this species.

Therefore, our goals were to identify REG-family members, particularly of the REG1 and REG3 sub-families, in dogs, both on a genomic and proteomic level, to set the groundwork for the investigations of these proteins as potential markers for canine pancreatitis and sepsis.

## Materials and methods

### Annotation of the canine REG protein family

Genomic sequences were derived from the National Center for Biotechnology Information (NCBI (RRID: SCR_006472)^1^ The human reference genome GRCh38.p13 assembly and NCBI annotation release 109 were used. The domestic dog (*Canis lupus familiaris*) assembly GCF_014441545.1 (ROS_Cfam_1.0; NCBI annotation release 106) was searched for homologous sequences using NCBI Genome Data Viewer and NCBI BLAST (RRID:SCR_004870). Additionally, the publicly available databases Universal Protein Resource (Uniprot; RRID:SCR_002380)^2^, and Ensembl (RRID:SCR_002344)^3^ were searched for predicted genes and proteins of the REG family in dogs. Genomic regions were analyzed for repetitive sequences with RepeatMasker (RRID:SCR_012954)^4^, and dot plots were performed using PipMaker and MultiPipMaker (RRID:SCR_011806)^5^ Individual human exons were aligned to canine genomic regions to identify coding sequences (CDS), including start and stop codons and exon-intron boundaries. The potential canine homologous cDNA and protein sequences were subsequently aligned to known human, mouse (assembly GRCm39; NCBI annotation release 109), and rat (assembly mRatBN7.2; NCBI annotation release 108) REG genes and proteins using Lalign^6^, Clustal Omega (RRID:SCR_001591)^7^ and NCBI BLAST for sequence comparison. Obtained sequences were submitted to the Vertebrate Gene Nomenclature Committee^8^ for official designation. In the following, these new names are used, despite the next annotation release containing these updates not yet having been published at the time of writing this manuscript. Canine RNA sequencing (RNA-Seq) data was downloaded from BarkBase^9^ (32), an open-access dog epigenomic resource containing RNA-Seq and Assay for Transposase-Accessible Chromatin using sequencing data of several organs of adult and fetal canine tissue. Annotated canine transcripts were compared to RNA-Seq data using the Integrative Genomics Viewer (RRID:SCR_011793)^10^ (33) to search for evidence of transcription of the identified potential canine REG genes. Trimmed mean of M values were extracted from the available binary alignment map files.

### Biological samples

Formalin-fixed, paraffin-embedded histological tissue sections were obtained from the Institute of Animal Pathology of the Vetsuisse Faculty, University of Bern, Switzerland. These included healthy pancreatic tissue collected from the cadaver of a 4-year-old male entire Dachshund submitted for *postmortem* examination after sudden death attributed to heart failure, and pancreatic biopsies from a 12-year-old male castrated Yorkshire terrier with spontaneously occurring acute pancreatitis. Both were submitted to the Institute of Animal Pathology as part of the clinical workup at the owners' request.

Tissue for pancreatic homogenates was harvested from an 8-year-old male castrated Labrador retriever, submitted for *postmortem* examination with signed owner consent after humane euthanasia for neoplastic disease not involving the pancreas. Homogenates were prepared using snap-frozen pancreas tissue stored at −80°C. Tissue (25 mg) was homogenized in 500 μl homogenization buffer (50 mM Tris/HCl pH 8, 10 mM NaCl, 1mM EDTA) with addition of a proteinase inhibitor cocktail (cOmplete^TM^ Mini EDTA-free; Roche, Cat#11836170001) on ice using a handheld homogenizer (VWR pellet mixer, VWR; Cat#431–0100). Samples were sonicated (Branson digital sonifier W-450 D, Branson Ultrasonics) for 3x 10s at 10%, centrifuged for 10 min at 2370 rcf and 4°C (Hettich Universal 320R, Hettich, Switzerland, Cat#1401), and the pellets discarded. Protein concentration was determined in the supernatant diluted in Pierce 660 nm Protein Assay reagent (Thermo Scientific, Cat#22660) using a Nanodrop 2000 Spectrophotometer (Thermo scientific, Cat#ND-2000), against a pre-diluted bovine serum albumin standard curve (Thermo Scientific, Cat#23208).

Canine plasma was collected from leftover blood submitted to the Diagnostic Laboratory of the Vetsuisse Faculty, University of Bern, Switzerland. Blood was taken as part of routine diagnostic workup of a client-owned 6-year-old female spayed miniature Australian shepherd admitted to the Small Animal Hospital of the Vetsuisse Faculty, University of Bern, Switzerland with spontaneously occurring acute hemorrhagic diarrhea syndrome with secondary sepsis. Therefore, ethical approval was waived by the local ethics committee, but signed owner consent for use of leftover biological material was obtained. Plasma from a healthy control dog (3-year-old male intact Irish wolfhound) was collected as part of the Small Animal Hospital's blood donor program. Samples were stored for up to 5 days at +5°C, up to 1 month at −20°C, and up to 2.5 years at −80°C.

### Recombinant proteins and antibodies

Recombinant human REG1A and REG3A proteins, produced in *Pichia pastoris* culture, and antibodies raised in rabbits and guinea pigs against human recombinant REG1A and REG3A as previously described (6, 34, 35) were obtained from the Pancreas Research Laboratory, University of Zürich, Switzerland.

Recombinant canine REG3E protein spanning AA 28–176 was produced in Chinese hamster ovary cells by a commercial laboratory^11^.

### Immunohistochemistry

Immunohistochemistry was performed at the Pancreas Research Laboratory of the University of Zürich, using a previously established protocol (35, 36). Briefly, sections of formalin-fixed, paraffin-embedded pancreatic tissue were deparaffinised, boiled in citrate for antigen demasking, and blocked prior to incubation with the primary antibody directed against human REG1A or REG3A, respectively, at a 1:800 dilution. Following incubation with the biotinylated secondary antibody (Dako Cat# P0141), avidin and biotinylated enzyme (Vectastain ABC kit, Vector Laboratories, Cat#PK-4000), sections were stained with 3,3-diaminobenzidine tetrahydrochloride dihydrate, counterstained with hematoxylin (Dako, Cat#CS70030-2), and coverslipped.

### Western blot

Canine plasma corresponding to 200–500 μg of total protein was denatured with 1:10 β-mercaptoethanol (Sigma, Cat#M3148) in Bolt^TM^ 4x LDS Sample Buffer (Novex life technologies, Cat#B0007) for 5 min at 95°C. Proteins were separated on an acrylamide gel (Bolt 10% Bis-Tris Plus, Invitrogen, Cat#NW00100BOX) and blotted onto a polyvinylidene fluoride (PVDF) membrane (Trans-Blot Turbo Mini 0.2 μm PVDF Transfer Packs, BioRad, Cat#1704156). After blocking for 3 h in 5% Bovine Serum Albumin (BSA; Sigma, Cat#A3059), in 0.2% TTBS (Tween 20, Sigma, Cat#P9416; Tris-Buffered Saline 20x solution ultrapure, Thermo Scientific, Cat#J75892) or in 5% milk (Rapilait, Migros) in TTBS, the membrane was incubated over night at 4°C with the primary antibodies (anti-human REG1A and REG3A) at a 1:1000 dilution in 2% BSA-TTBS or milk-TTBS, and for 2 h with a secondary anti-IgG horseradish peroxidase-coupled antibody (Dako Cat# P0141; Cell Signaling Technology Cat# 7074, RRID:AB_2099233) at a 1:5000 and 1:2000 dilution, respectively. Bands were detected using a Vilber Fusion FX (Witec AG) after 5 min incubation with the chemiluminescent substrate (SuperSignal West Pico PLUS Chemiluminescent Substrate, Thermo Scientific, Cat#34577).

### Protein sequencing

For protein analysis, canine pancreas homogenates (40 μg total protein) and plasma (500 μg total protein) from one dog, previously identified as having a strong positive signal of the appropriate length in Western blot, were separated and blotted onto a PVDF membrane as described above. Membranes were stained with Coomassie, and the area corresponding approximately to the 17 kDa REG protein band was cut out and placed in an Eppendorf tube without additives. Protein analysis and interpretation were performed at the Proteomics and Mass Spectrometry Core Facility at the Department for BioMedical Research of the University of Bern, Switzerland using liquid chromatography and mass spectrometry. The mass spectrometry proteomics data, including detailed sample and data processing protocols have been deposited to the ProteomeXchange Consortium^12^ *via* the PRIDE (37) partner repository with the dataset identifier PXD035694.

### Trypsin digestion

Trypsin digestion of 15 μg recombinant protein was performed in 150 μl of Tris-calcium buffer with the addition of 0.5 μg TPCK-treated bovine trypsin (Sigma-Aldrich Cat#T1426) for 30 min at 37°C, as previously described (14).

## Results

### Annotation of the canine REG protein family

**Figure 1:**
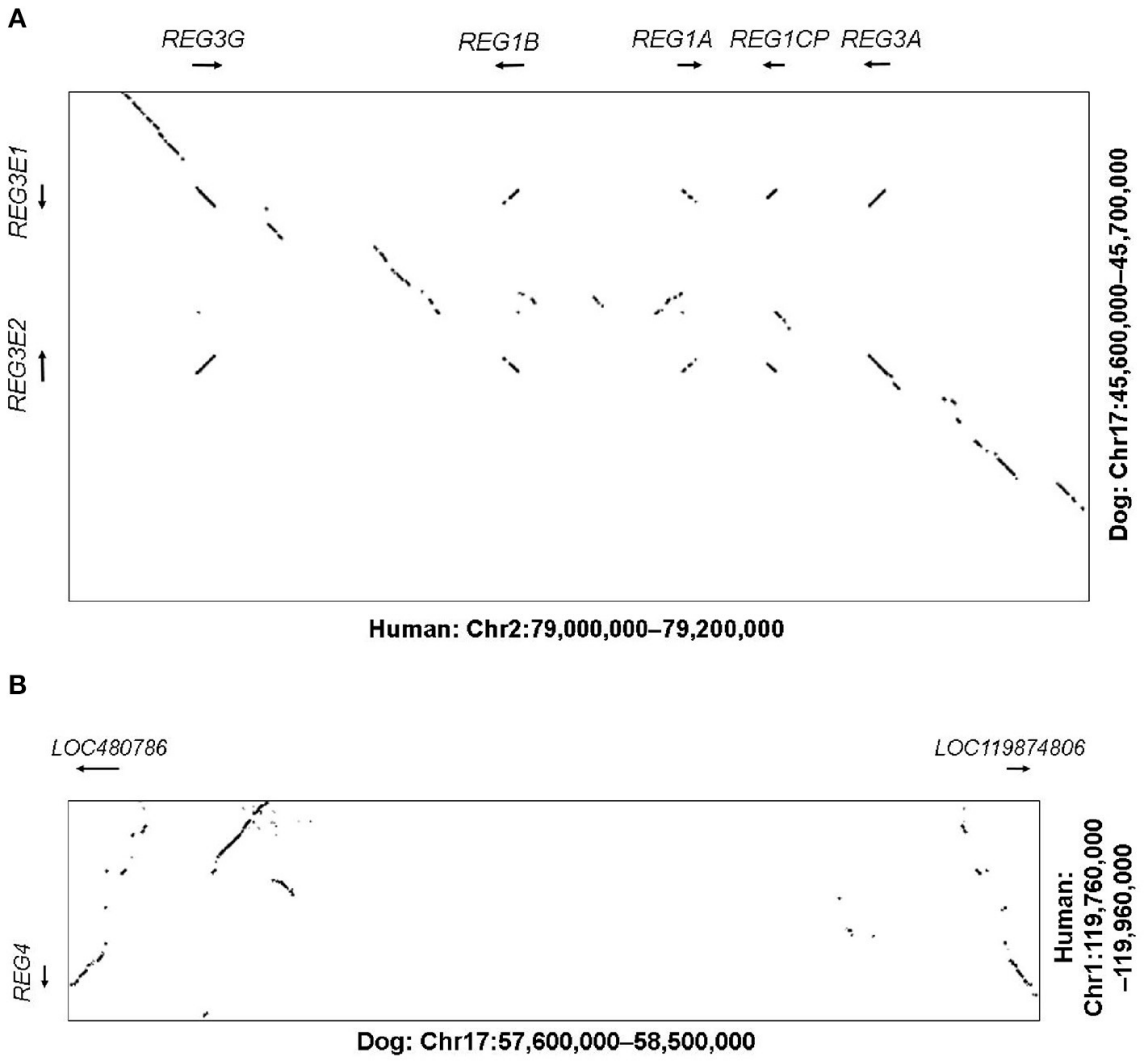
**(A)** Dot plot illustrating the comparative analysis of the *REG3* genomic region. The human region containing the *REG1* and *REG3* subfamilies (Chr2:79.0-79.2M) is shown on the X-axis against the corresponding canine region (Chr17:45.6–45.7M) on the Y-axis. Sequences of high similarity between both species are depicted as diagonal lines at the corresponding positions, representing potential homologous genes, with the direction of the line reflecting the direction of the genomic sequences (forward vs. reverse strand). Whilst the human region contains two *REG1* and two *REG3* genes plus a pseudogene, the canine region only contains two *REG3* genes, namely *REG3E1* and *REG3E2*. **(B)** Dot plot illustrating the comparative analysis of the *REG4* genomic region. The human region containing *REG4* (Chr1:119.76–119.96M) on the Y-axis is shown against the corresponding canine region (Chr17:57.6–58.5M) on the X-axis. Whilst in people, only one *REG4* gene is present, two canine candidate *REG4* genes were identified (*LOC480786* and *LOC119874806*).

In humans, the genes *REG1A, REG1B, REG3A, REG3G* as well as the pseudogene *REG1CP*, are clustered in the region 79–79.2 Mb on chromosome 2p12, flanked by the well-conserved genes *LRRTM4* and *CTNNA2*. The corresponding syntenic region in the canine assembly ROS_Cfam_1.0 was found to include 45.6–45.7 Mb on chromosome 17. With help of NCBI BLAST and Dotplot, we identified two possible homologous genes in that region, *LOC403411* (also labeled *REG3A*) and *LOC100687463* (also labeled *REG3G-like*), on the reverse and forward strand, respectively (Figure 1A). These genes were subsequently designated *REG3E1* and *REG3E2* by the Vertebrate Gene Nomenclature Committee. Both genes had 6 exons. For *REG3E2*, different 5'-untranslated region transcript variants were proposed, namely *XM_038690416.1* (variant X1) and *XM_038690417.1* (variant X2), and *NM_001256535.2*, the latter of which lacked exon 1, but had a longer 5'- untranslated region in exon 2. The predicted CDSs of both *REG3E* candidate genes had 531 base pairs, shared 99.6% identity on CDS level, and translated to a 100% identical amino acid sequence, identified on Uniprot as F1PZP0_CANLF, and subsequently termed REG3E based on the Vertebrate Gene Nomenclature Committee nomenclature. The two differing nucleotides were at position c.348 (C in *REG3E1*, A in *REG3E2*) and c.468 (T in *REG3E1*, C in *REG3E2*).

In humans, the *REG4* gene is located separately on chromosome 1p12, between *HMGCS2* and *NOTCH2* (119.76–119.96 Mb), which matched region 57.6–57.7 Mb on canine chromosome 17. One candidate gene, namely *LOC480786*, was found in this region on the reverse strand, with a CDS of 477 base pairs and 6 exons. BLAST search revealed the presence of an additional canine *REG4* candidate, currently termed *LOC119874806*, approximately 800 kb further distal, at 58.5 Mb, in a head to head orientation with *LOC480786* (Figure 1B).

**Figure 2:**
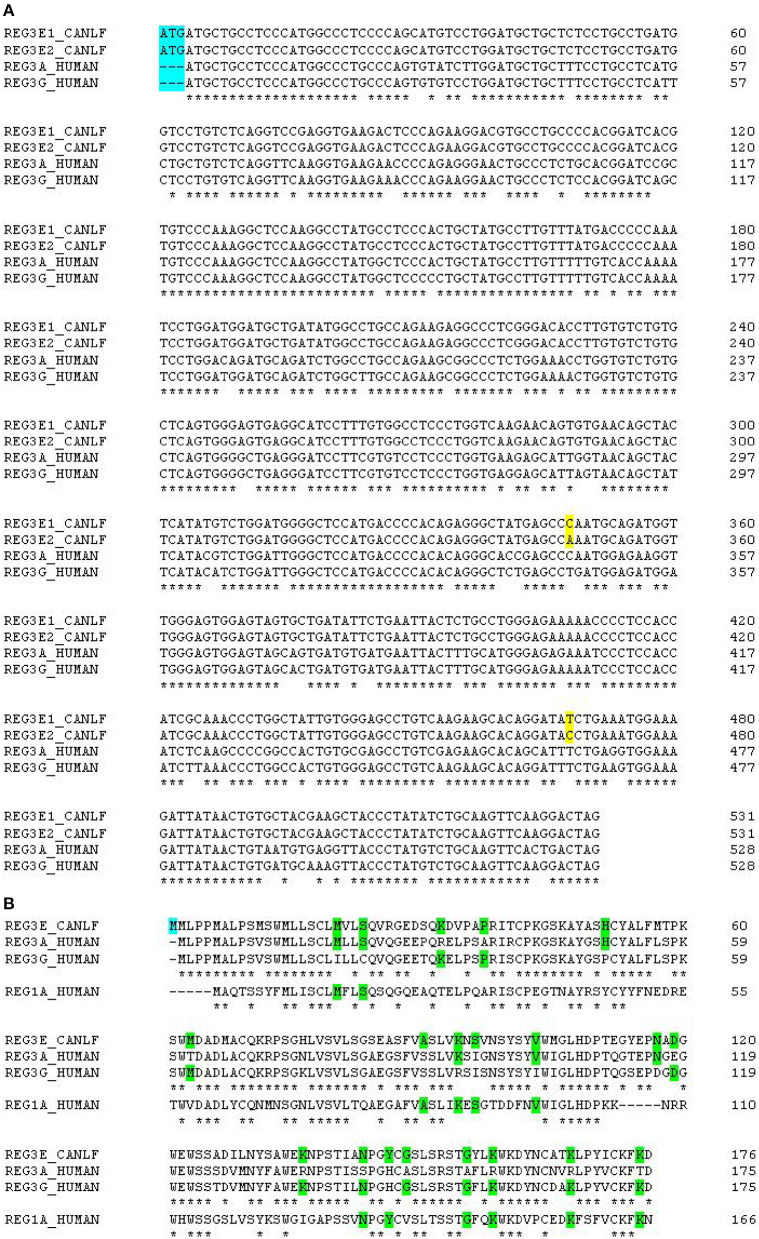
**(A)** Multiple alignments of canine and human *REG3* genes. Note the additional start codon in dogs, immediately before the start codon in the human genes (highlighted in blue). The two differing nucleotides between canine *REG3E1* and *REG3E2* are highlighted in yellow. Canine *REG3* genes have 84% identity score compared to human *REG3A* and 86% compared to human *REG3G*. **(B)** Multiple alignments of canine REG3E, human REG3A, REG3G and REG1A protein sequences. The canine predicted REG3E protein has an additional methionine at the N-terminus (highlighted in blue), 74 and 77% identity score compared to human REG3A and human REG3G, respectively, and 46% identity when compared to human REG1A. Stars under the first three lines indicate identity between all REG3 sequences; stars under the bottom line indicate identity between all four REG sequences. Amino acids common to some, but not all of the human sequences are highlighted in green.

Alignment scores of the CDS of these four candidate genes and their corresponding amino acid sequences compared to all members of the REG families in human, mouse, and rat are listed in Supplementary Table 1. In summary, canine *REG3E1* and *REG3E2* shared the highest similarity with human *REG3G*, with a cDNA identity of 86.0 and 85.5%, respectively, and with 77.1% identity on protein level (Figure 2). Compared to mice and rats, the CDS of both canine *REG3E* genes shared the highest similarity to *Reg3g* with 74.4–75% identity; on protein level, both had highest identities with Reg3b, namely 66.5% in comparison to rat and 64.7% in comparison to mouse. Both canine REG4 candidates shared highest identity with REG4 in all three examined species, with scores ranging from 73.8–83.6% identity for CDS, and from 58.9–9% on protein level. Lower similarities were identified between the canine REG candidates and REG1 in any species; CDS and protein identities were below 67 and 47%, respectively.

**Figure 3:**
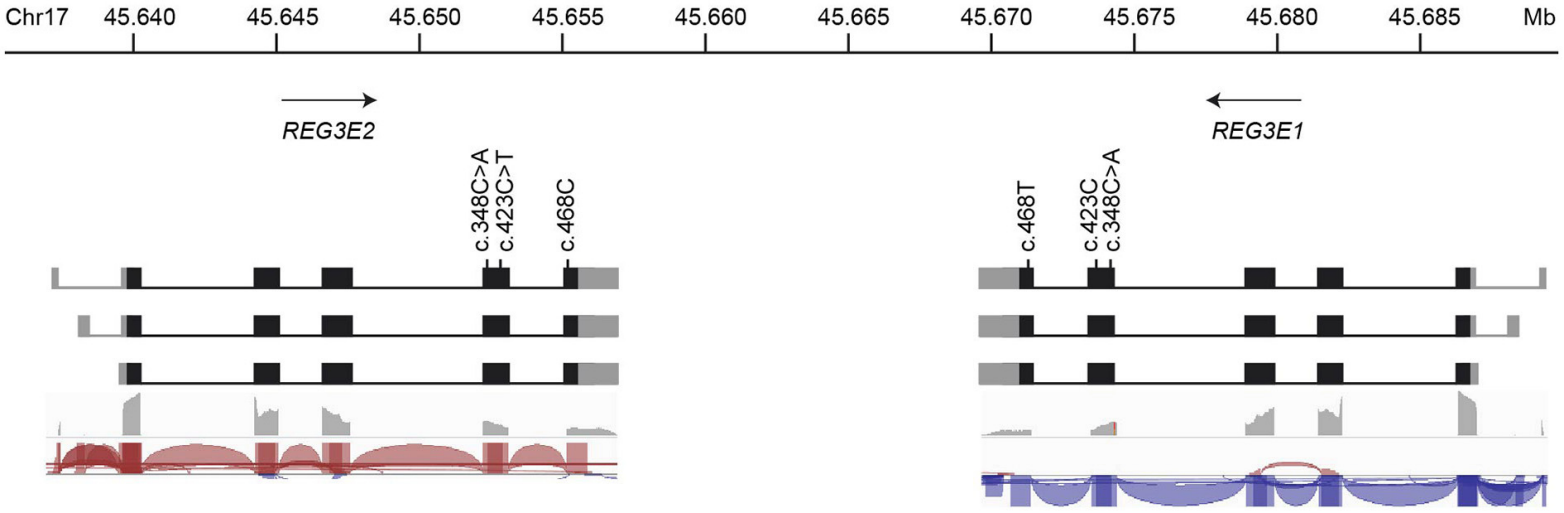
Schematic representation of the genomic organization, nucleotide variations, and transcript variants of *REG3E1* and *REG3E2*. The longest transcript isoforms (top row) are the most prevalent (>50%), whereas the shortest, lacking exon 1, are the least common (< 10%) for both genes. The bottom two rows represent sashimi plots from screenshots of the Integrative Genomics Viewer using RNA-Seq data from Barkbase. Note that due to the almost complete sequence identity between *REG3E1* and *REG3E2*, transcript reads cannot be mapped reliably to either gene.

There is experimental support for transcription of the canine *REG3E1* and *REG3E2* genes in RNA-Seq data available at Barkbase (Figure 3), but due to the almost complete sequence identity between *REG3E1* and *REG3E2*, transcript reads could not be mapped reliably to either gene. Of the two nucleotides differing between the *REG3E1* and *REG3E2* transcripts annotated on ROS_Cfam_1.0 assembly, only c.468 seemed to represent a true difference between the paralogous gene copies, while it was suspected that the A/C difference at position c.348 represents a single nucleotide variant (SNV) found in both genes and segregating in the dog population. Additionally, a SNV was found at c.423 of *REG3E2* (C>T) in RNA-Seq data of three dogs (Figure 3). The RNA-seq data provided supporting evidence for three transcript variants differing in their 5' untranslated regions for both genes (Fig 3), with the longest having most of the reads (>50%), and the shortest, which was lacking exon 1, being least prevalent (< 10%). All three variants had the same open reading frame. Trimmed mean of M values normalized counts for both *REG3E* gene transcripts together were highest in the pancreas, ranging from 1,699 to 69,950, exceeding expression in other tissues by 6 to over a million-fold in the same individual. Relatively high counts were also found in the cardiac atrium, the small intestine, and, in one dog, in the liver, with large variation between individuals (Supplementary Table 2). There was no evidence for transcription of either of the *REG4* candidates in the pancreas of any of the dogs.

### Immunohistochemistry

**Figure 4:**
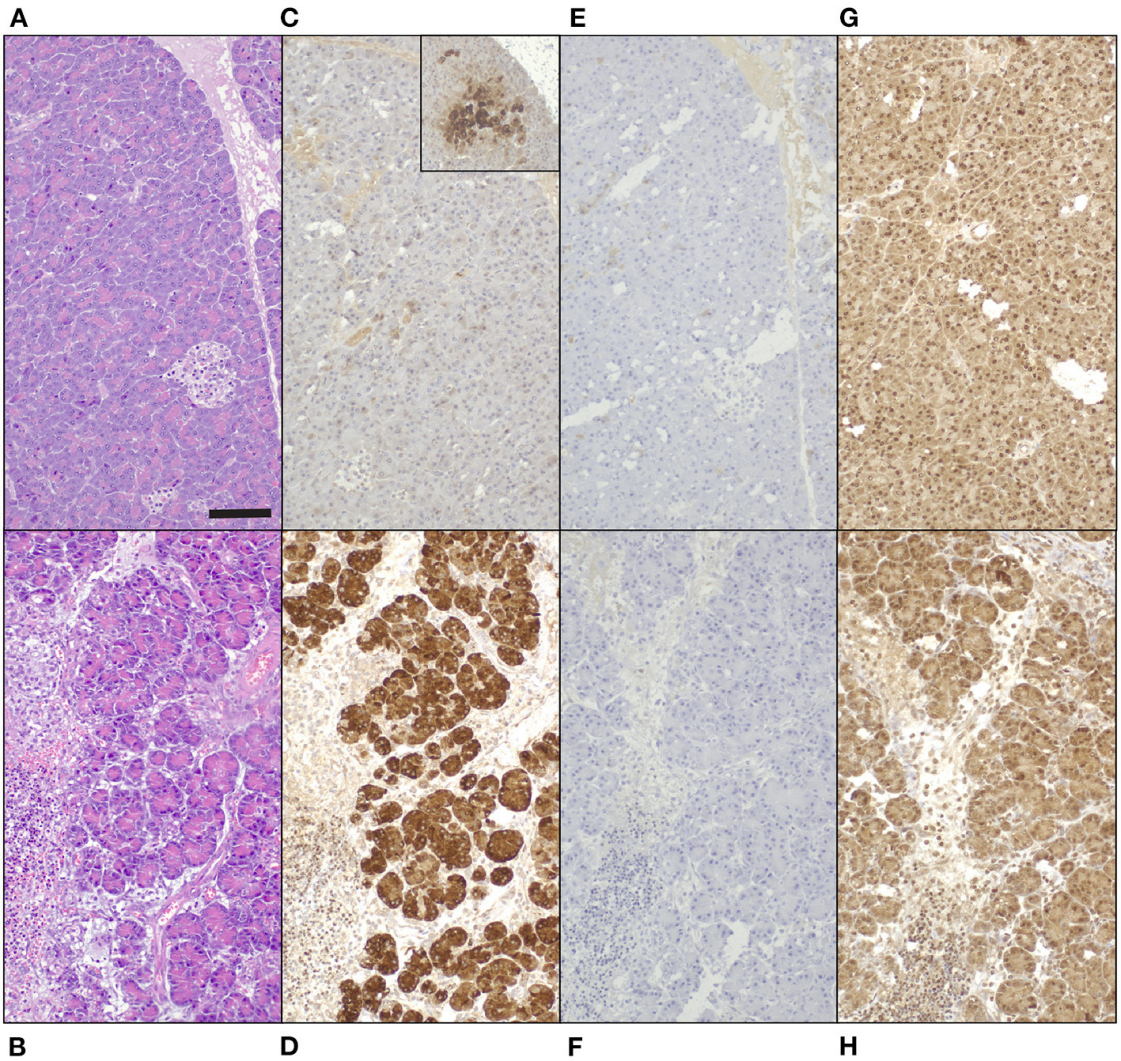
Tissue biopsies of normal canine pancreas [top row; **(A,C,E,G)**] and canine pancreas with acute pancreatitis [bottom row. **(B,D,F,H)**], 200x magnification, bar = 100 μm. **(A,B)** Hematoxylin and eosin. **(C-H)** 3,3-diaminobenzidine tetrahydrochloride dehydrate chromogen, hematoxylin counterstain. **(C,D)** Guinea pig anti-human REG3A antibodies produce minimal, patchy signal in healthy control tissue [**(C)** inset 100x magnification], and strong cytoplasmic immunoreactivity in pancreatic acinar cells in acute pancreatitis **(D). (E,F)** No signal in neither tissue with guinea pig anti-human REG1A antibodies. **(G,H)** Non-specific, mainly nuclear signal in both tissues to rabbit anti-human REG1A antibodies.

Immunohistochemical staining demonstrated a strong positive signal in pancreatic acinar cells against anti-human REG3A antibodies in inflamed pancreas, whereas only weak and patchy signal was observed in healthy control tissue (Figures 4A–D). Anti-human REG1A antibodies yielded no positive signal in any of the samples (Figures 4E–H).

### Western blot

**Figure 5:**
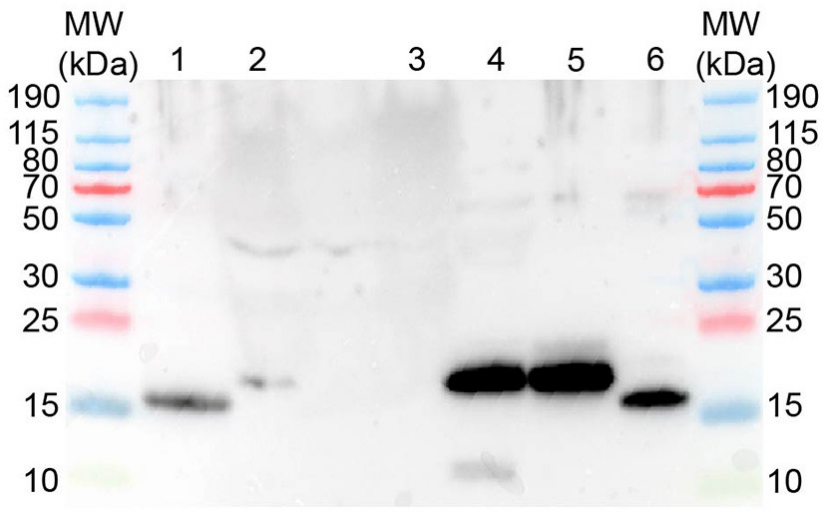
Western blot using rabbit anti-human REG3A antibodies yields protein bands of approx. 16-17 kDa. MW–Molecular weight marker; 1–human recombinant REG3A; 2–canine plasma (sepsis); 3–canine plasma (healthy control); 4–canine pancreas homogenate; 5–canine recombinant REG3E (untreated); 6–canine recombinant REG3E (trypsin digested).

Western blot with anti-human REG3A antibodies against pancreatic homogenates from a healthy canine pancreas, and plasma from a dog suffering from sepsis and acute hemorrhagic diarrhea syndrome yielded a positive signal of approx. 17 kDa, while plasma from a healthy control dog did not reveal any positive signal (Figure 5). Western blot using two different anti-human REG1A antibodies against canine plasma did not result in any immunoreactivity (data not shown).

### Protein characterization

**Figure 6:**
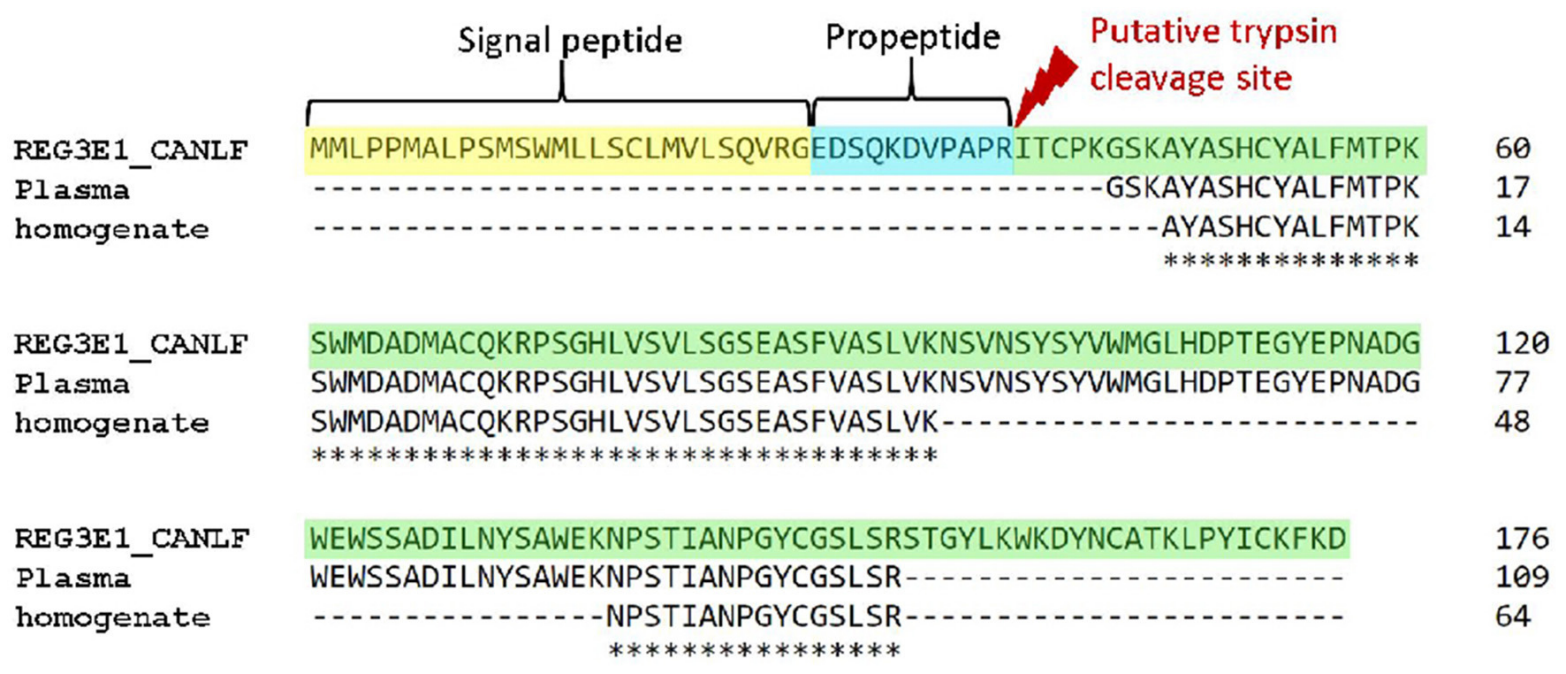
Alignments of canine REG3E protein with protein sequences obtained from canine plasma and pancreas homogenates with mass spectrometry. N-terminal sequences corresponding to the human signal peptide and propeptide are highlighted in yellow and blue, respectively.

Mass spectrometry of canine plasma from a dog suffering from sepsis and acute hemorrhagic diarrhea syndrome blotted onto PVDF membrane yielded a sequence of 109AA, corresponding to position 44–152 of REG3E. Analysis of canine pancreas homogenates detected two sequences, spanning AA 47–94 and AA 137–152 of the same protein (Figure 6). These sequences had 100% identity with the canine protein sequence derived from Uniprot, and no significant overlap with any other proteins recorded in the interrogated databases.

Incubation of the recombinant canine REG3E protein (molecular weight 17.4 kDa due to the inserted human rhinovirus 3C cleavage site) with trypsin demonstrated a visible reduction in molecular size corresponding to the calculated difference of 1.3 kDa, as evidenced by Western blot (Figure 5).

## Discussion

Our data provide evidence for the existence of a member of the REG protein family in dogs. This protein, named REG3E, is expressed from two closely related genes, *REG3E1* and *REG3E2*. It is synthesized in the canine pancreas, and was found in plasma.

Compared to humans and rodents, for whom REG genes and proteins have been thoroughly investigated, dogs appear to lack members of the so far described REG family (15). Most importantly, we found no evidence for the presence of a homolog to REG1 in dogs, either on a genomic, proteomic, or on immunologic level, and therefore conclude that REG1 is unlikely to exist in dogs. We cannot fully exclude that *REG1* genes in dogs may have been missed due to gaps in the canine assembly. However, the absence of a potential homolog of REG1 in our proteomic analysis of canine pancreas and plasma, and the lack of cross-reactivity with different anti-human REG1A antibodies in different assays further support the absence of this protein in dogs. Given the phylogenetic relationship in the placental mammalian evolutionary tree placing rodents and humans in a different superordinal clade (Supraprimates aka Euarchontoglires) than dogs (Laurasiatheria) (38), it could be speculated that REG1 genes arose through duplication within this clade, as was previously proposed for other members of the REG family (39). Alternatively, REG1 genes may have been lost through deletion in the sister clade Laurasiatheria. Further investigations into the REG gene and protein family in other mammalian species would be needed to further establish the evolution of this gene family in different mammals.

Based on the sequence identity of over 40% between human REG1 proteins and canine REG3E, and overlapping functions of REG1 and REG3 proteins in humans and rodents, it would be plausible that canine REG3E assumes some of the functions attributed to REG1 in other species (40). Conversely, it is also possible that there is no functional counterpart to REG1 in the dog, as it has been demonstrated that *Reg1*^−/−^ knockout mice develop normally, concluding that Reg1 may be dispensable for survival in mice (41). As the biological roles of REG proteins have not been fully established, it is impossible to infer specific functions between REG homologs in different species. Ongoing advances in the functional characterization of REG proteins may help to further establish the significance of the apparent absence of REG1 in dogs.

Spontaneously occurring pancreatic stones, where REG1A was first discovered as PSP in humans (9), are extremely rare in dogs, despite pancreatitis being a relatively common disease in this species (42). In experimental settings using dogs, administration of ethanol lead to protein plug formation in pancreatic juice (43), and to chronic pancreatitis with pancreatic stone formation when combined with pancreatic duct ligation (44), but no insoluble fraction corresponding to precipitated REG1A could be found in such plugs (43). Therefore, since REG1A is the main component of pancreatic stones in people (9), it is possible that the paucity of pancreatic stone formation in dogs is at least in part due to the suspected absence of REG1 in this species.

The canine REG3E protein identified in this study had a slightly higher sequence identity with human REG3G than REG3A (PAP) (77 vs. 74%), and is most similar to Reg3b and Reg3g in both rats and mice. In order to avoid misinterpretation as orthologs, the designations *REG3E1* and *REG3E2* were assigned to the canine genes by the Vertebrate Gene Nomenclature Committee. Given previous entries on NCBI and Ensembl designating *REG3E1* as *REG3A*, it is reasonable to assume that O'Reilly and colleagues have in fact identified REG3E in canine feces (31). Similar to reports in humans, where REG3A is upregulated in inflammatory bowel disease (19), the afore mentioned study found increased amounts of REG3A in the feces of dogs with chronic enteropathies compared to healthy controls, suggesting that this protein could potentially be of use in the diagnosis of canine intestinal disorders.

Analysis of RNA-Seq data derived from Barkbase (32) revealed marked differences in expression of *REG3E* RNA between tissues, with the highest number of reads found in the pancreas, followed by the cardiac atrium and the small intestine. In humans and rodents, REG1A protein is predominantly produced in the pancreas, whereas REG3A is primarily found in the small intestine (35). This is paralleled by findings in lambs, where a “PAP-like protein” mRNA was expressed 300-fold higher in ileal and jejunal Peyer's patches compared to the pancreas (30). Therefore, finding the highest *REG3E* transcript abundance in the canine pancreas is somewhat unexpected, and could suggest that this protein compensates for the lack of a REG1 homolog in dogs. In rodents, REG proteins were found to be expressed both in Paneth cells as well as enterocytes, predominantly in the duodenum for REG1 and in the ileum for REG3 (21, 35). Such precise anatomic localization was not possible for canine *REG3E* mRNA expression in our study, as the available RNA-Seq data only included one sample from the small intestine for each individual (32). While high mRNA levels do not linearly translate into high protein expression, likely due to posttranscriptional, translational, and posttranslational regulation amongst other factors, there is nonetheless a degree of correlation between both (45, 46), suggesting that protein expression of REG3E is likely also highest in the pancreas, intestine, and cardiac atrium.

The high mRNA expression found in the cardiac atrium was surprising, exceeding that of the small intestine in most sampled individuals. To date, no mRNA or protein expression has been demonstrated in cardiac tissue in humans for either REG1A or REG3A (the human protein atlas; HPA; RRID:SCR_006710)^13^. Furthermore, expression of Reg proteins in cardiac tissue was minimal in rats and mice with induced septic events (35). However, the cardiac atrium was not specifically sampled in the aforementioned study, therefore it is possible that their results are solely reflective of Reg protein expression in cardiac ventricular tissue, and high expression in the atria could have been missed. Nonetheless, the significance of high *REG3E* gene transcription in the canine cardiac atrium cannot be extrapolated from current knowledge in other species.

The difference in gene expression between individual dogs was also noteworthy, with an approximately 40-fold difference between the dog with the highest and the lowest expression of *REG3E* in pancreatic tissue. Unfortunately, the disease status and reason for euthanasia of most dogs donated to the Barkbase project are not known, other than neoplastic disease being an exclusion criterion. Quantification of REG3E mRNA or protein in different tissues from dogs with selected diseases known to induce production of REG proteins in other species, such as pancreatitis, sepsis, or gastrointestinal disease, would be needed for a more meaningful interpretation of gene expression patterns.

Immunohistochemistry using anti-human REG3A antibodies showed a very strong immunoreactivity throughout pancreatic sections of a dog suffering from acute pancreatitis, but minimal, patchy immunoreactivity in histologically normal canine pancreas. These findings confirm that a member of the REG3 subfamily is produced in pancreatic tissue of dogs, and suggests that it is preferentially expressed during pancreatitis, similar to rats (36, 47). The positive signal for REG3 appeared to be limited to acinar cells of the exocrine pancreas in the examined dog. This is in keeping with previous investigations localizing both Reg1a and Reg3a to pancreatic acinar cells in rats (36, 47). Conversely, no immunoreactivity for Reg1a or Reg3a was found in islets of these rats in either study. This stands in contrast to the first report of *Reg1* cDNA and mRNA, isolated from regenerating pancreatic islet of 90% depancreatized rats, giving this protein family its name of regenerating island-derived proteins (10).

Since REG proteins have been identified in the pancreatic juice of several species (9, 27, 47), they are expected to be produced in the exocrine secretory portion of the organ. Examination of REG3E protein expression in tissue sections from a larger cohort of dogs with diseases such as pancreatitis and sepsis, ideally coupled with measurements of protein concentration in blood and pancreatic juice, may help elucidate the kinetics of REG proteins in diseased dogs in the future.

We were able to sequence large portions of the REG3E protein by mass spectrometric analysis both in canine plasma and pancreatic homogenates. Despite being incomplete, the obtained fragments were identical to the protein sequence predicted from genomic annotation and corresponded to sequences published on Uniprot, allowing us to identify the protein unequivocally as REG3E. We hypothesize that the missing amino acids are most likely a result of the digestion process, creating larger hydrophobic fragments, which were lost in the mass spectrometer during analysis due to their electrochemical properties. It is very unlikely that these fragments are truly missing from the native protein, as we separated the proteins in the plasma and tissue samples electrophoretically, and specifically only selected the area corresponding to the protein band of approximately 16–17 kDa for proteomic analysis.

We could demonstrate that recombinant canine REG3E protein undergoes cleavage of a small fragment when exposed to trypsin *in vitro*, visibly reducing protein size. This parallels findings in humans and rodents, where several members of the REG family have been shown to lose an undecapeptide by proteolytic cleavage at the N-terminus (14, 48, 49). It is reasonable to assume that the canine REG3E protein possesses the same cleavage site due to conserved amino acid sequences at this position and comparable reduction in protein band size on Western blot. However, N-terminal sequencing of the truncated proteins would be necessary to confirm this hypothesis with certainty. The biological significance of this cleavage has not been fully unveiled, but there is evidence for polymerization of the trimmed REG protein to insoluble fibrils in several species including humans, rodents, and cattle. Additionally, it has been shown that REG proteins only un-fold their full bactericidal activity, mediated by the formation of hexameric transmembrane pores, after cleavage of the N-terminal propeptide, presumably to inhibit cytotoxic effects on epithelial cells (22, 49).

Our study had some limitations in addition to those discussed above, the main one being the lack of availability of plasma and pancreatic tissue from the same individuals. However, the primary goal of this work was to provide evidence for the existence of REG proteins in dogs, which could be achieved with the current experimental design. Further studies will be needed to examine REG3E expression in different tissues of dogs affected by local and systemic inflammatory conditions to establish the dynamics of REG3E in canine disease.

In conclusion, we demonstrated on the genomic, proteomic, and immunologic levels the existence of REG3E in dogs, setting the groundwork for further investigations into this protein in this species. Especially the high expression in pancreas and small intestine, which appeared to be more abundant in dogs with acute pancreatitis, sepsis and gastrointestinal disease, emphasizes the potential value of REG3E as biomarker for these diseases in dogs.

## Data availability statement

The data presented in the study are deposited in the ProteomeXchange Consortium repository http://www.proteomexchange.org/, accession number PXD035694.

## Ethics statement

Ethical review and approval was not required for the animal study because only leftover biological material was used, taken for routine diagnostic workup of client-owned dogs with naturally occurring disease or enrolled in a blood-donor program, with informed owner consent. Written informed consent was obtained from the owners for the use of leftover material in this study.

## Author contributions

LP, JH, MM, RG, and TR contributed to conception and design of the study. JH and TR provided scientific supervision. TL, MM, and RG provided scientific advice. LP and TR provided biological samples and materials, and performed laboratory experiments. LP wrote the first draft of the manuscript. LP, TL, RG, and TR designed the figures. All authors contributed to manuscript revision, read, and approved the submitted version.

## Funding

This study was supported by the initiator grant of the Stiftung Tierspital of the Vetsuisse Faculty Bern, Switzerland.

## Conflict of interest

Author RG is the inventor of an assay covered by patent no: EP 2185937 B2 method for assaying sepsis in humans, which is owned by the University of Zürich (Zürich, Switzerland).

The remaining authors declare that the research was conducted in the absence of any commercial or financial relationships that could be construed as a potential conflict of interest.

## Publisher's note

All claims expressed in this article are solely those of the authors and do not necessarily represent those of their affiliated organizations, or those of the publisher, the editors and the reviewers. Any product that may be evaluated in this article, or claim that may be made by its manufacturer, is not guaranteed or endorsed by the publisher.

## Footnotes

^1^ https://www.ncbi.nlm.nih.gov/

^2^ https://www.uniprot.org/

^3^ https://www.ensembl.org/

^4^ http://www.repeatmasker.org/

^5^ http://pipmaker.bx.psu.edu/

^6^ https://embnet.vital-it.ch/software/LALIGN_form.html

^7^ https://www.ebi.ac.uk/Tools/msa/clustalo/

^8^ https://vertebrate.genenames.org/

^9^ www.barkbase.org

^10^ https://software.broadinstitute.org/software/igv

^11^ https://www.biotem-antibody.com/

^12^ http://www.proteomexchange.org/

^13^ www.proteinatlas.org

## Abbreviations

CDS: Coding sequence
NCBI: National Center for Biotechnology Information
PAP: Pancreatitis-associated protein
PSP: Pancreatic stone protein
PVDF: Polyvinylidene fluoride
REG: Regenerating islet-derived protein
RNA-Seq: RNA sequencing
SNV: Single nucleotide variant.
