# Supplementary material for: Identification of regenerating island-derived protein 3E in dogs
Source: Front Vet Sci. 2022 Oct 28;9:1010809. doi: 10.3389/fvets.2022.1010809 (PMC9650133; doi:10.3389/fvets.2022.1010809)
Supplement: Supplementary file 1 [file Data_Sheet_1.docx]

**Supplementary material**

**Supplementary table 1:** Alignments of cDNA and protein sequences of canine REG candidates with human, rat, and mouse sequences. All numbers expressed in % identity.

| Canis lupus familiaris (ROS_Cfam_1.0) | | Homo sapiens sapiens (GRCh38.p13) | | | | | | | |
| --- | --- | --- | --- | --- | --- | --- | --- | --- | --- |
|  |  | REG1A | REG1B |  | REG3A |  |  | REG3G | REG4 |
| REG3E1 (LOC403411) | CDS  Protein | 66.3  46.2 | 65.2  45.6 |  | 84.1  74.3 |  |  | 86.0  77.1 | 50.8  28.7 |
| REG3E2  (LOC100687463) | CDS  Protein | 66.5  46.2 | 65.4  45.6 |  | 83.7  74.3 |  |  | 85.8  77.1 | 51.2  28.7 |
| LOC480786 | CDS  Protein | 53.9  34.3 | 55.2  36.6 |  | 51.4  29.6 |  |  | 51.4  27.4 | 83.6  74.7 |
| LOC119874806 | CDS  Protein | 52.5  32.8 | 54.4  35.1 |  | 51.7  29.0 |  |  | 49.4  26.2 | 83.2  75.9 |
|  | | Rattus norvegicus (mRatBN7.2) | | | | | | | |
|  | | REG1A |  |  | REG3A | REG3B |  | REG3G | REG4 |
| REG3E1 (LOC403411) | CDS  Protein | 61.9  46.0 |  |  | 72.4  58.0 | 72.9  66.5 |  | 74.8  66.1 | 53.5  31.6 |
| REG3E2  (LOC100687463) | CDS  Protein | 62.1  46.0 |  |  | 72.0  58.0 | 72.5  66.5 |  | 74.4  66.1 | 53.3  31.6 |
| LOC480786 | CDS  Protein | 56.2  35.8 |  |  | 50.8  35.5 | 53.8  31.1 |  | 51.5  32.5 | 73.8  58.9 |
| LOC119874806 | CDS  Protein | 54.1  34.3 |  |  | 51.0  34.5 | 55.4  31.1 |  | 50.8  31.8 | 74.1  61.4 |
|  | | Mus musculus (GRCm39) | | | | | | | |
|  | | REG1 |  | REG2 | REG3A | REG3B | REG3D | REG3G | REG4 |
| REG3E1 (LOC403411) | CDS  Protein | 63.4  46.6 |  | 61.2  43.5 | 74.8  63.2 | 73.9  64.7 | 68.7  52.3 | 75.0  64.4 | 53.7  31.6 |
| REG3E2  (LOC100687463) | CDS  Protein | 63.6  46.6 |  | 61.3  43.5 | 74.4  63.2 | 73.5  64.7 | 68.3  52.3 | 74.6  64.4 | 53.2  31.6 |
| LOC480786 | CDS  Protein | 56.0  35.1 |  | 53.8  33.6 | 50.8  34.5 | 53.1  32.5 | 52.7  28.1 | 52.0  32.1 | 74.3  59.9 |
| LOC119874806 | CDS  Protein | 54.8  33.6 |  | 53.2  32.1 | 50.4  33.1 | 52.9  32.9 | 53.2  28.1 | 51.7  31.5 | 74.1  63.1 |

**Supplementary table 2:** RNA-Seq data from Barkbase; Transcription of REG3 genes expressed as trimmed mean of M values in various tissues of five adult dogs (N/A; no data for this organ available in this individual)

| Organ | **Dog 1** | **Dog 2** | **Dog 3** | **Dog 4** | **Dog 5** |
| --- | --- | --- | --- | --- | --- |
| **Adipose** | 1029.6 | 0 | 0 | 94.5 | 0.2 |
| **Adrenal** | 31.3 | 0 | 0 | 0 | 0 |
| **Bladder** | 3.7 | 0 | 0 | 0 | 0 |
| **Bone marrow** | 0.1 | 0 | 0 | 0 | 0.6 |
| **Cartilage** | 0.2 | 0.1 | N/A | 0 | 0.3 |
| **Cerebellum** | 0.3 | 0 | 0.1 | 0.4 | 0.2 |
| **Colon** | 5.7 | 1.0 | 1.2 | 1.2 | 0.2 |
| **Occipital cortex** | 0.4 | 0.1 | 0.3 | 3.7 | 0.4 |
| **Frontal cortex** | 0.4 | 0 | 0 | 0 | 0.4 |
| **Kidney cortex** | 2.0 | 0.1 | 0 | 0 | 20.1 |
| **Kidney medulla** | 5.5 | 0 | 88.1 | 3.7 | 0.4 |
| **Left atrium** | 259.7 | 73.8 | 3.6 | 0 | 1661.9 |
| **Left ventricle** | 0.6 | 0.4 | 0.4 | 0 | 0.1 |
| **Liver** | 4365.9 | 0.1 | 0 | 0 | 3.1 |
| **Lung** | 18.3 | 0 | 0 | 0.3 | 820.0 |
| **Lymph node** | 3.3 | 0 | 0.1 | 0.2 | 2.8 |
| **Pancreas** | 69950.5 | 1699.1 | 5602.8 | 5500.2 | 9717.7 |
| **Pituitary** | 8.6 | 0 | N/A | 0 | 0.1 |
| **Right atrium** | 197.9 | 113.5 | 2.2 | 97.9 | N/A |
| **Right ventricle** | 0.3 | 0.1 | 0.1 | 0.6 | 4.5 |
| **Salivary** | 0.1 | 0 | 0 | 0 | 0.1 |
| **Skeletal muscle** | 1.8 | 0 | 1.7 | 0.1 | 2.0 |
| **Skin** | 0.6 | 2.1 | 0 | 0 | 0.1 |
| **Small intestine** | 36.3 | 35.4 | 120.7 | 29.0 | N/A |
| **Spleen** | 59.1 | 0 | 0.3 | 0 | 0.6 |
| **Stomach** | 43.8 | 0 | 0 | 0 | 0.3 |
| **Thyroid** | 0.5 | 0 | 0 | 1.8 | N/A |
